# Combined dual-channel fluorescence depth sensing of indocyanine green and protoporphyrin IX kinetics in subcutaneous murine tumors

**DOI:** 10.1117/1.JBO.30.S1.S13709

**Published:** 2024-11-18

**Authors:** Madhusudan B. Kulkarni, Matthew S. Reed, Xu Cao, Héctor A. García, Marien I. Ochoa, Shudong Jiang, Tayyaba Hasan, Marvin M. Doyley, Brian W. Pogue

**Affiliations:** a University of Wisconsin-Madison, Department of Medical Physics, Madison, Wisconsin, United States; b CIFICEN (UNCPBA - CICPBA - CONICET), Tandil, Buenos Aires, Argentina; c Dartmouth College, Thayer School of Engineering, Hanover, New Hampshire, United States; d Massachusetts General Hospital, Harvard Medical School, Boston, Massachusetts, United States; e University of Rochester, Department of Electrical and Computer Engineering, Rochester, New York, United States

**Keywords:** tissues, fluorescence, depth sensing, fiber optics, kinetics, cancer

## Abstract

**Significance:**

Fluorescence sensing within tissue is an effective tool for tissue characterization; however, the modality and geometry of the image acquisition can alter the observed signal.

**Aim:**

We introduce a novel optical fiber-based system capable of measuring two fluorescent contrast agents through 2 cm of tissue with simple passive electronic switching between the excitation light, simultaneously acquiring fluorescence and excitation data. The goal was to quantify indocyanine green (ICG) and protoporphyrin IX (PpIX) within tissue, and the sampling method was compared with wide-field surface imaging to contrast the value of deep sensing versus surface imaging.

**Approach:**

This was achieved by choosing filters for specific wavelengths that were mutually exclusive between ICG and PpIX and coupling these filters to two separate detectors, which allows for direct swapping of the excitation and emission channels by switching the on-time of each excitation laser between 780- and 633-nm wavelengths.

**Results:**

This system was compared with two non-contact surface imaging systems for both ICG and PpIX, which revealed that the fluorescence depth sensing system was superior in its ability to resolve kinetics differences in deeper tissues that would normally be dominated by strong signals from skin and other surface tissues. Specifically, the system was tested using pancreatic adenocarcinoma tumors injected into murine models, which were imaged at several time points throughout tumor growth to its ∼6-mm diameter. This demonstrated the system’s capability to track longitudinal changes in ICG and PpIX kinetics that result from tumor growth and development, with larger tumors showing sluggish uptake and clearance of ICG, which was not observable with surface imaging. Similarly, PpIX was quantified, which showed slower kinetics over different time points, and was further compared with the wide-filed imager. These results were further validated through depth measurements in tissue phantoms and model-based interpretation.

**Conclusion:**

This fluorescence depth sensing system can be used to sample the interior blood flow characteristics by ICG sensing of tissue as deep as 20 mm into the tissue with sensitivity to kinetics that are superior to surface imaging and may be combined with other imaging modalities such as ultrasound to provide guided deep fluorescence measurements.

## 1 Introduction

Biomedical optical sensing can be used to extract multidimensional molecular and functional information from tissues when combined with appropriate reporter molecules. Within this area, fluorescence sensing provides one of the most specific strategies for high-sensitivity information about tissue, and there are several dyes available for use.^1^^–^^3^ The most commonly used human reporters are in the near-infrared (NIR) and red wavelength bands due to high penetration through deep tissue.^4^ In this study, a prototype system was developed that could passively switch between two fluorophores to measure fluorescent signals through sub-surface tissue volumes in a small probe-style geometry. In addition, the system was designed to achieve deep tissue sampling, allowing for measurement well below the tissue surface. Both of these are described below along with the prototype design. The fluorescent agent most widely utilized in humans is indocyanine green (ICG), a perfusion probe tracer for assessing tissue vascular function,^5^ which exhibits peak fluorescence emission at around 830 nm and peak absorption at roughly 780 nm. ICG-aided diagnosis has become a common practice in certain neurovascular and gastrointestinal surgeries and for general tissue perfusion/function assessment.^6^ In addition, the next most commonly used agent is protoporphyrin IX (PpIX), a molecule universally produced in all living organisms and present in all living cells, with an endogenous production that is stimulated *in vivo* through the administration of the PpIX precursor molecule 5-aminolevulinic acid (5-ALA).^7^^,^^8^ PpIX produces a reasonably bright red-NIR fluorescence at wavelengths 635 to 720 nm, which can be imaged superficially with blue light excitation at 405 nm or more deeply with red 635-nm excitation. The ability to image both of these human-use fluorophores with the same system was part of the goal of our prototype fluorescence depth sampling system.^9^ The development of a system that could simultaneously measure these two fluorophores could have an inherent value because the ICG signal indicates vascular flow/perfusion, whereas the PpIX signal indicates the metabolic function of heme synthesis. Both of these signals have unique relevance to tumors and their signal changes with tumor growth and response to therapies.^9^^–^^11^

One of the subtler but tricky aspects of fluorescence measurement is that the signal is strongly affected by the geometry of the tissue and the light illumination and detection patterns. Surgical use has been widely adopted with non-contact camera systems that image with planar excitation illumination and planar optical imaging of the emission.^12^ This works well logistically for surgical imaging but inherently limits the signal to the most superficial layers of the tissue, such as 1- to 3-mm deep. In comparison, deeper sampling is possible but comes at the cost of using a separated source-detector geometry and higher attenuation that comes with the light transport through tissue.^13^^–^^15^

This study introduces a novel optical fiber-based system capable of measuring fluorescent contrast agents in deep tissues. By simultaneously acquiring fluorescence and excitation data, we can accurately determine both ICG and PpIX within deep tissue. Furthermore, this describes the development of a fiber optic probe-based dual-channel fluorescence/excitation fluorescence depth sensing system consisting of source-detector pairs used for fluorescence detection using contrast agents under different experimental conditions. The system was designed for sequential ICG and PpIX sensing in a manner that can couple to other imaging tools, such as an ultrasound transducer. Furthermore, the approach of measuring the fluorescence-to-excitation (F/E) ratio minimizes fiber coupling–related issues and tissue optics attenuation factors.^16^ As a proof-of-concept, this developed prototype system was validated for *in vivo* studies implemented on mice to see the sensitivity and stability of the system that showed the potential change across both the fast ICG kinetics and the slower PpIX signals, to differentiate tumor tissue as it grows and as compared with normal tissue. A comparison of the signals is shown with planar surgical surface imaging systems to compare the value in sensing tumor tissue under the thin mouse skin.

## 2 Materials and Methods

### 2.1 System Development

A single-channel depth sensing system was constructed using components for fiber coupling to maximize light efficiency for fluorescent sensing, to measure porphyrin production and ICG flow kinetics. The system hardware comprised a source-detector pair setup with electronic control and acquisition from the detector to the computer via Labview software. The source unit had two laser modules: an FC-coupled fiber optic–driven 780-nm laser diode with a maximum 80-mW average power (model: TECIRL-80G-780-TTL-A, World-StarTech, Toronto, Canada) for ICG imaging and an FC-coupled fiber optic–driven 635-nm laser diode with a maximum 50-mW average power (model: TECRL-50G-635-TTL-A, World-StarTech, Toronto, Canada) for PpIX imaging, respectively. Here, the 780-nm laser was directly controlled by the USB communication port of the computer for pulsed laser modulation and triggering using control commands executed in the NI-VISA function via LabVIEW. The 635-nm laser was pulsed modulated and triggered by using TTL modulation via analog output pins of the NI DAQ board. Herein, the square wave–based pulsating signal was generated from this laser, and signal detection was based on the amplitude of the modulated signal at a desired frequency. This was coupled to a modified 200-μm diameter, 0.22 NA, fiber optic cable with FC-connector (Model: M122L05, Thorlabs, Newton, New Jersey, United States), which had the tissue contact end custom-made by gluing on a polylactic acid (PLA) filament-based 3D-printed (Creator 3 Pro, FlashForge, Zhejiang Flashforge 3D technology Co., Zhejiang, China) cylindrical tube to the cut fiber and polishing for a small round ferrule of 3-mm diameter, providing the necessary rigidity for positioning during imaging studies.

**Fig. 1:**
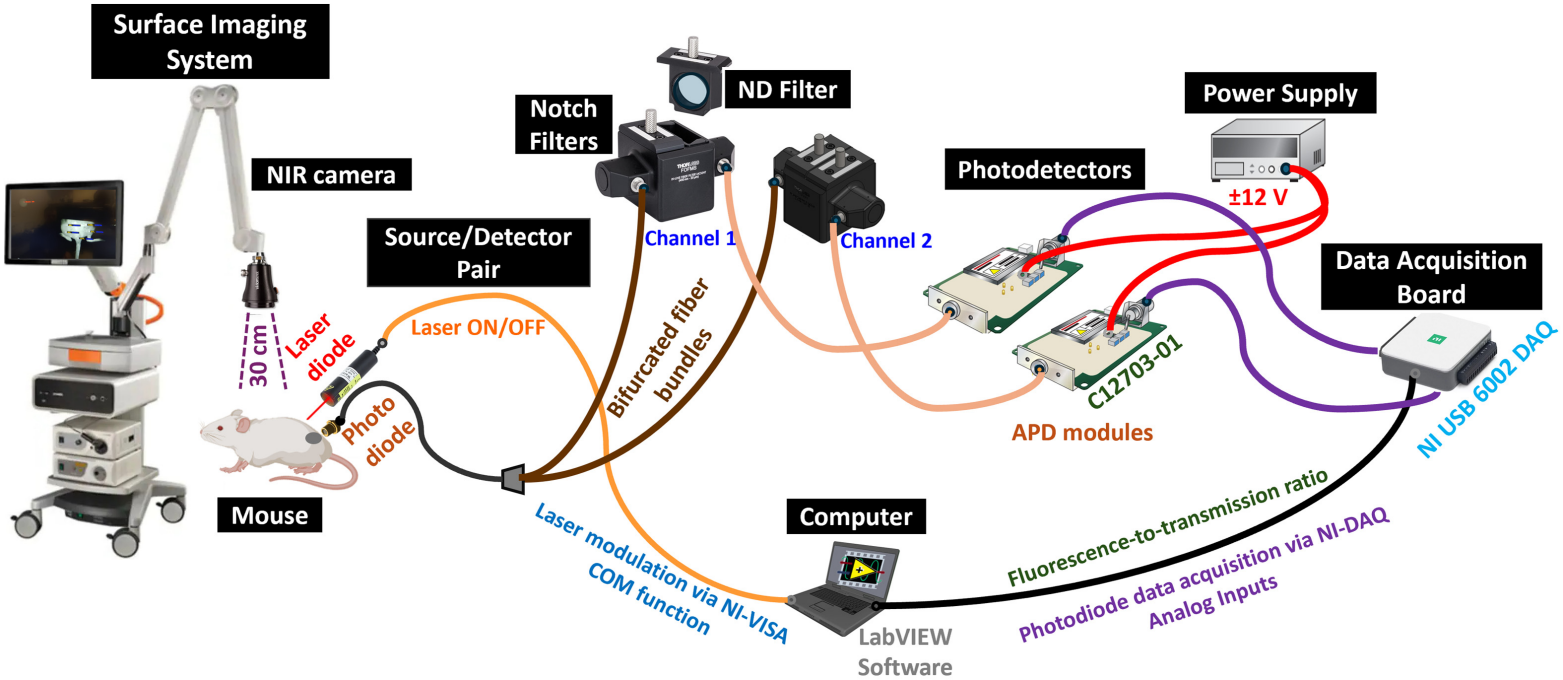
Schematic representation of the prototype system hardware showing individual components including lasers, detectors, optical filters, and data acquisition devices. Imaging with a surface imaging system was performed in parallel in the same mice for comparison.

The detector end included a splitter fiber to split the light signal into two channels, for detection of the excitation light and the fluorescent light. Each detector was an avalanche photodiode module, with the light first going through a notch filter block. The SMA-coupled bifurcated fiber bundle had 19 fibers in the Bundle with Ø200  μm, SMA coupling, and 2-m length (BF19Y2LS02, Thorlabs). The excitation channel included a notch filter that blocked the PpIX excitation light and passed just the ICG excitation light (NF633-25 - Ø25 mm Notch, Thorlabs), as well as a neutral density filter (ND10B, Thorlabs) to prevent saturation of the excitation channel. The emission channel passed the fluorescence from ICG with a notch filter (NF785-33 - Ø25 mm Notch, Thorlabs) to block excitation light. Two avalanche photodiodes were used as a photodetector (C12703-01, Hamamatsu Corp, Bridgewater, New Jersey, United States) for capturing the fluorescence-to-excitation ratio simultaneously. APD modules were powered by ±12  V (E3630A 35 W, Keysight Tech, Santa Rosa, California, United States). The output of the APD module was fed to a DAQ I/O card to transform the acquired intensity values to a voltage signal, (NI-USB DAQ 6002, 50  KS/s sampling rate, 16-bit resolution, NI, Austin, Texas, United States) measured by LabVIEW software (Version 2022 Q3 (64-bit) 22.3.1f8). Figure 1 illustrates the schematic representation of the prototype system hardware showing individual components, including source/detector pair, optical filters, power supply, and data acquisition.

### 2.2 Protocol for Animal Studies

Animal studies were conducted in accordance with protocols approved by the University of Wisconsin School of Medicine and Public Health Institutional Animal Care and Use Committee (Protocol M006554). All efforts were made to minimize animal suffering. All surgical procedures and ICG injections were performed under isoflurane anesthesia.

Four (n=4) Athymic Nude Mice (cat # 6905M, Envigo, Indianapolis, Indiana, United States) 6 to 8 weeks of age were placed on a low-chlorophyll diet to minimize tissue autofluorescence. Mice were anesthetized using a SomnoSuite Low-Flow Anesthesia system (Kent Scientific, Torrington, Connecticut, United States). A 3% isoflurane concentration was used for induction, and a 2% to 2.5% concentration was used for maintenance, using a flow rate of 500  mL/min and 100% oxygen as the carrier gas. Once anesthetized, each mouse received a 0.1-mL injection containing $1\times 10^{6}$ cells into the right flank.

To examine how the signal changes with tumor growth, mice were imaged on various days after implantation. ICG (cat # 099555 Matrix Scientific, Columbia, South Carolina, United States) was suspended in sterile distilled water and diluted to a concentration of 0.7  mg/mL. Mice were individually placed in a prone position on a sheet of opaque black plastic with an infrared warming pad underneath, which was connected to and monitored with SomnoSuite’s onboard warming system. A surgical plane of anesthesia was verified via toe pinch, and the hind legs of each mouse were secured to the imaging bed using 3 M Transpore medical tape to avoid excessive movement during imaging.

For ICG imaging, mice were given a retro-orbital injection of 1  mg/kg ICG for fast IV uptake and continuously imaged for 10 min thereafter. The procedure for injecting ICG and measuring with the probe for 10 min was conducted before tumor implantation for each mouse and subsequently on multiple separate days after the tumor reached 3 mm in diameter (once the tumor location became visibly distinguishable in diameter); similarly, this was followed by the next few days after, where the tumor growth size reached ∼6  mm in diameter. ICG was freshly prepared on the day of each injection, and the same dose was injected each time.

For PpIX imaging, mice were given a retro-orbital injection of 250  mg/kg aminolevulinic acid (ALA) in sterile saline, the pro-drug contrast that develops PpIX over the course of several hours.^17^ The imaging was limited to being done with just a few hourly time points as the mice had to be re-anesthetized during each imaging session.

### 2.3 Procedure for Tumor Cell Line Development

AsPC-1 pancreatic adenocarcinoma cells were obtained from the American Type Culture Collection (ATCC) and grown in Roswell Park Memorial Institute (RPMI) 1640 Media supplemented with 10% fetal bovine serum and 1% penicillin/streptomycin. These were stored in a 5% ${\mathrm{CO}}_{2}$ incubator. For injection, cells were re-suspended in a 50/50 mixture of PBS and Corning Matrigel (cat # CB-40234A, Fisher Scientific, Hampton, New Hampshire, United States), drawn up into U100 insulin syringes, and placed on ice until injection.

## 3 Phantom Experiments

### 3.1 Phantom Mimicking and Testing Using ICG and PpIX Fluorophores

To assess the expected signal magnitudes and transient responses during imaging beyond superficial tissues, the fluorescence depth sensing system was first validated for the fluorescence-driven signal to determine the linearity range using different concentrations such as 0  μM as a control, followed by 0.1, 0.2, 0.3, 0.5, 1, and 2  μM of ICG and PpIX tissue phantom mimicking solutions. The recipe used for the preparation of the phantom solution includes 1% intralipid (Sigma Aldrich, St. Louis, Missouri, United States) and 0.1% ink (Carbon Black Pigment, Speedball Art Products, Statesville, North Carolina, United States) diluted in deionized water added with different ICG concentrations and, similarly, 2% tween diluted in deionized water added with different PpIX concentrations.^18^ The excitation signal specifies a means to standardize variations in laser intensity and tissue variations simultaneously during the imaging of the fluorescence signal. The fluorescence-to-excitation ratio was calculated based on the data points obtained from the fluorescence and excitation channels that displayed a linear response tracking the fluorescence signal. Furthermore, the fluorescence-to-excitation ratio offers equalization against fluctuations in laser or excitation intensity. Herein, the significance of the fluorescence-to-excitation ratio can be traced back to the origins of the born normalization strategy introduced by Ntziachristos and Weissleder in 2001 in context to fluorescence image reconstruction.^19^ Furthermore, this study can be utilized to correct the fluctuations that persisted in the input signal due to several different reasons, for instance, laser power fluctuating over time or imperfect fiber contact on tissue surface due to motion artifacts. In tomographic approaches, the F/E ratio is particularly more useful because the sources may be subject to different powers and the detector to different gains, and thus, the measured raw signal at each detector may not be directly comparable if no normalization is performed.

**Fig. 2:**
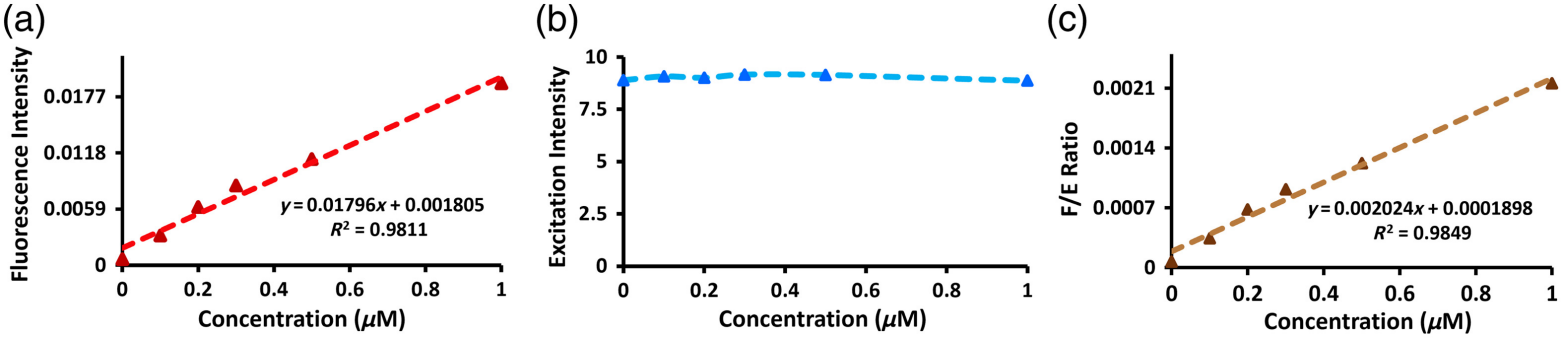
Fluorescence intensity (a), laser excitation intensity (b), and fluorescence-to-excitation ratio (c) were measured in tissue-mimicking phantoms with varying ICG concentrations between 0 and 1  μM. Both the fluorescence and fluorescence-to-excitation ratio showed stable linearity within these concentrations, whereas the excitation intensity was largely independent of the fluorophore concentration.

Figure 2 illustrates the different ICG concentration studies ranging from control (0  μM), 0.1, 0.2, 0.3, 0.5, and 1  μM to observe the increase in fluorescence signal. Although the fluorescence signal was low, it showed a linear response with incremental concentration levels, as shown in Fig. 2(a). The excitation signal was steady and independent of the fluorophore, as shown in Fig. 2(b). Furthermore, the fluorescence-to-excitation ratio, depicted in Fig. 2(c), showed a linear behavior with the ICG concentration.

**Fig. 3:**
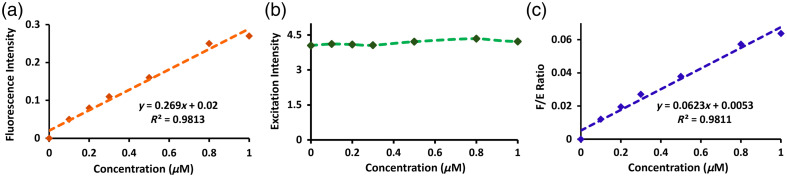
Fluorescence intensity (a), laser excitation intensity (b), and fluorescence-to-excitation ratio (c) were measured in tissue-mimicking phantoms with varying PPIX concentrations between 0 and 1  μM. Similar to the ICG measurements in Fig. 2, both the fluorescence and fluorescence-to-excitation ratio showed stable linearity within these concentrations, whereas the excitation intensity was largely independent of the fluorophore concentration.

Similarly, Fig. 3 illustrates the different PpIX concentration studies ranging from control (0  μM), 0.1, 0.2, 0.3, 0.5, and 1  μM. Figure 3(a) shows the linear fluorescence response. Figure 3(b) represents the excitation signal, which is independent of the fluorophore and appears to be steady. Figure 3(c) shows the fluorescence-to-excitation ratio, showing a linear response as well.

Finally, it can be noted that the F/E ratio strategy implemented here does not necessarily improve the stability of the signal either in Fig. 2 or 3; however, the current sensing system presented in this work is an intermediate step toward a more complex fluorescence tomography system,^20^ so, as stated above, the F/E approach will definitely have a more important effect. In any case, the most vital results in this study are that (i) the excitation channel is independent of the concentration and (ii) the linear behavior of the fluorescence signal remains linear after normalizing.

### 3.2 Fluorescence-Based Tissue Depth Sensing

**Fig. 4:**
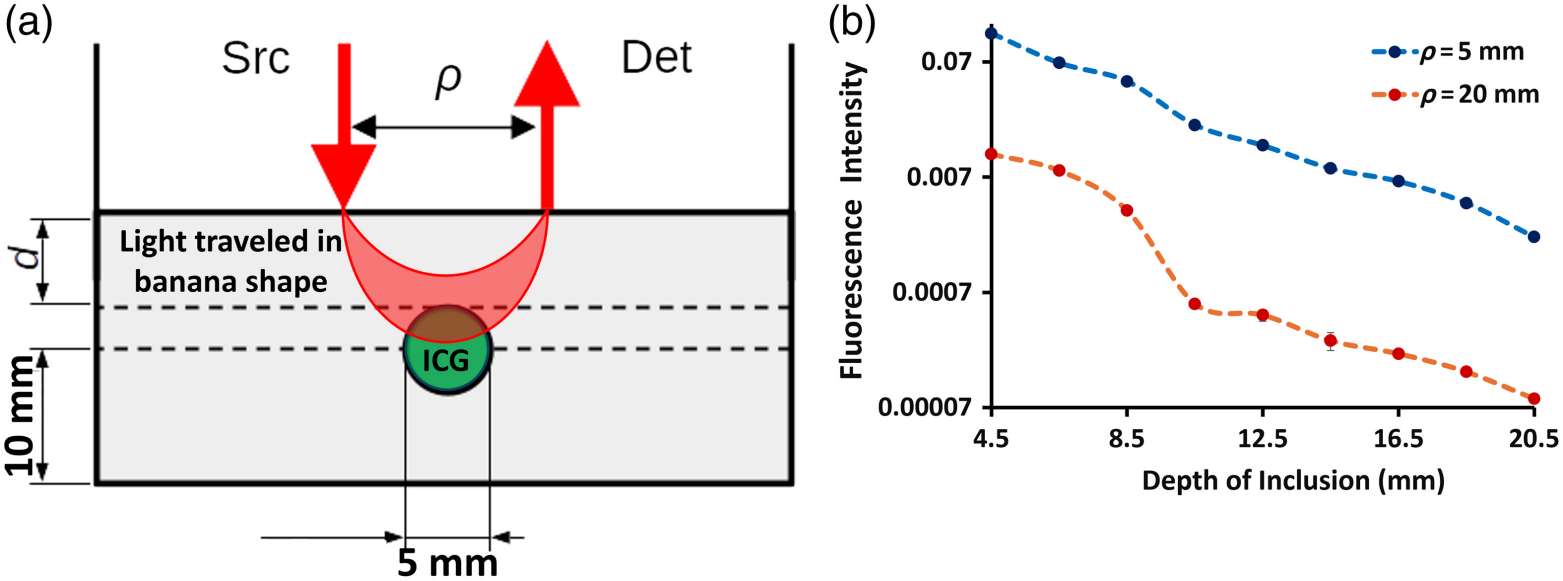
(a) Scheme of the depth analysis experimental setup using a spacing ρ between the laser source (Src) and the detector (Det), which was measured using a tube of ICG submerged in an intralipid-based solution. (b) The log of the fluorescence intensity was plotted as a function of the inclusion’s depth for ρ=5  mm (blue) and ρ=20  mm (red).

The sensitivity to depth of the fluorescence depth sensing system introduced in this work was carried out following an experimental setup, as schemed in Fig. 4(a). A cylindrical vial (diameter of 5 mm and length of 10 mm) was filled with a liquid solution of 1-μM ICG in deionized water. This vial was placed inside a plastic container ($10\times 14\times 60\;{\mathrm{cm}}^{3}$) with a liquid phantom made of deionized water, 1% Intralipid, and 0.1% India Ink; this proportion aimed at achieving typical living tissues optical properties [i.e., absorption coefficient $\mu_{aM}=0.01\;{\mathrm{mm}}^{-1}$ and reduced scattering coefficient $\mu_{s^{\prime }}=1\;{\mathrm{mm}}^{-1}$ (Ref. 21)]. The vial was suspended at 10 mm above the bottom surface of the container using a thin white thread; this method helped place the vial in a fixed position without floating, sinking, or displacing it in the lateral directions.^22^ The depth, d, of the vial was modified from 4.5 to 20.5 mm by adding liquid on top of it, and the fluorescence and excitation signals were measured with the source and detector fibers in contact with the free surface of the liquid phantom; in addition, the detector fiber was positioned on one side of the vial and the detector fiber on the opposite side, being the source–detector distances used in this study ρ=5  mm and ρ=20  mm. Finally, the system was isolated from ambient light by placing it inside a sealed black plastic box.

From Fig. 4(b), it can be seen that both sets of measurements (ρ=5  mm, blue; and ρ=20  mm, red) show a decrease in the fluorescence signal with the vial’s depth; naturally, the shortest source–detector distance measured higher intensities than the largest source–detector distance for the full depth range (with differences of more than one order of magnitude). The experimental data for ρ=20  mm present an abrupt change of slope at depth values between 8.5 and 10.5 mm; this may indicate that the photon paths begin to intercept the inclusion at this depth. This behavior is indicative of a change in optical properties sensed by the fluorescence depth sensing system.

A more thorough analysis can be performed by fitting the data shown in Fig. 4(b) with an appropriate theory describing fluorescence in turbid media. To this end, we used the analytical model introduced by Patterson and Pogue^24^ for the continuous-wave (CW) fluorescence signal [equation (13) of the referenced paper]:

$$F(\rho )=\frac{\mu_{af}\eta }{\mu_{aM}^{x}-\mu_{aM}^{m}}[R_{m}(\rho )-R_{x}(\rho )],$$ (1)

where the photon densities $N_{x}$ (at the excitation wavelength $\lambda_{x}$) and $N_{m}$ (at the emission wavelength $\lambda_{m}$) from the cited reference were replaced by the corresponding diffuse reflectances $R_{x}$ and $R_{m}$ (a procedure justified in that same paper); in addition, $\mu_{aM}^{x}$ and $\mu_{aM}^{m}$ represent the absorption coefficient of the medium at $\lambda_{x}$ and $\lambda_{m}$, respectively, whereas $\mu_{af}$ is the absorption coefficient of the fluorophore, and η is the quantum yield. Then, explicit expressions for $R_{x}$ and $R_{m}$ were taken from the paper by Farrel et al.^23^ to feed Eq. (1), with two additional simplifying assumptions:

1. The zero boundary condition was used instead of the extrapolated boundary condition.
2. The absorption coefficient of the medium was taken to be independent of the wavelength, i.e., $\mu_{aM}^{x}=\mu_{aM}^{m}$.

With the help of these two assumptions, it is possible to derive a fairly simple analytical expression to fit the data measured shown in Fig. 4(b). First, we can explicitly write $\mu_{aM}^{x}=\mu_{aM}^{x}$ ($\lambda_{x}$) and $\mu_{aM}^{m}=\mu_{aM}^{m}(\lambda_{m})$. As the fluorescence process requires that $\lambda_{m}>\lambda_{x}$, $\mu_{aM}^{m}$ can be rewritten as $\mu_{aM}^{m}=\mu_{aM}^{x}+\Delta \mu_{aM}$, where the sign of $\Delta \mu_{aM}$ depends on the absorption spectrum of the fluorophore in the range $\left[\lambda_{x},\lambda_{m}\right]$. With all this in mind, we can take the limit of Eq. (1) for $\Delta \mu_{aM}\to 0$:

$$\underset{\Delta \mu_{aM}\to 0}{\lim}\;\frac{\mu_{af}\eta }{\Delta \mu_{aM}}[R(\rho ,\mu_{aM}^{x}+\Delta \mu_{aM})-R(\rho ,\mu_{aM}^{x})]=-\mu_{af}\eta \frac{\partial R(\rho ,\mu_{aM})}{\partial \mu_{aM}};$$ (2)

the minus sign in the last member is needed to account for the fact that the detected diffuse reflectance R decreases (increases) with an increase (decrease) in $\mu_{aM}$. Now, we can derive equation (15) from Ref. 23 to reach the following expression for the fluorescence:

$$F(\rho )=\frac{C\;\exp[-\kappa \sqrt{\rho^{2}+z_{0}^{2}}]}{\sqrt{\rho^{2}+z_{0}^{2}}},$$ (3)

where $\kappa ={\left(3\mu_{aM}{\mu_{s}}^{\prime }\right)}^{-1/2}$ is the effective attenuation coefficient of the medium, $z_{0}=1/\mu_{s^{\prime }}$, and $C=3\mu_{af}\eta /4\pi$. Equation (3) was used to fit the data shown in Fig. 4(b) at the two source–detector separations and for each depth of the inclusion, using κ and C as fitting parameters.

**Fig. 5:**
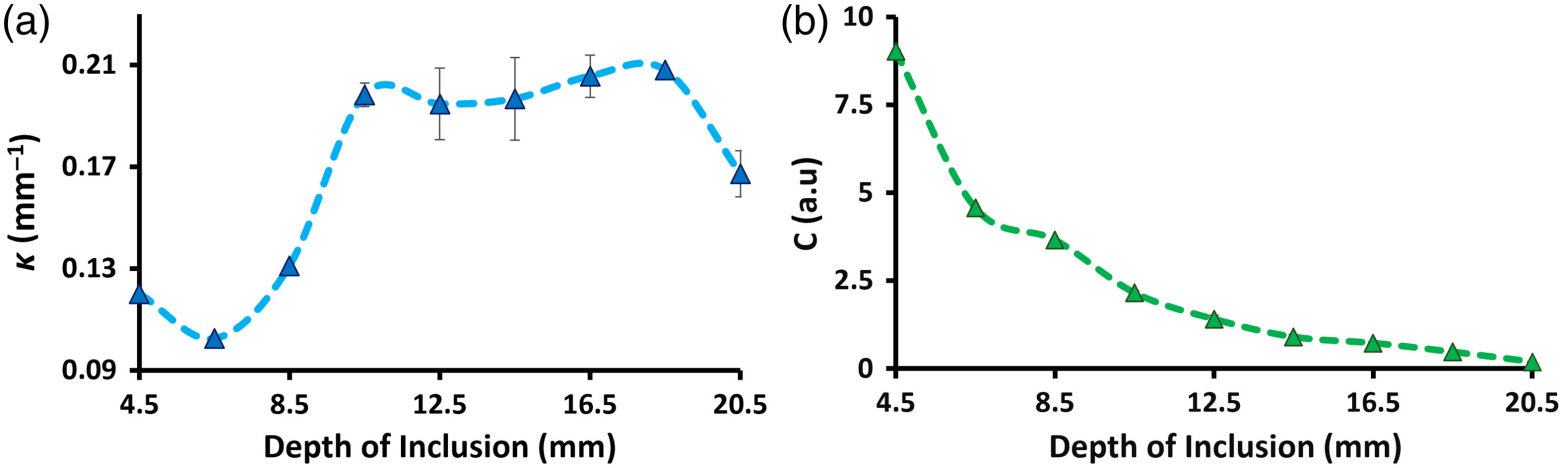
Results from the fitting process using the model given by Eq. (1): (a) effective attenuation coefficient, κ; (b) amplitude factor, C.

Figure 5 summarizes the results from the fitting process. Panel (a) shows the retrieved effective attenuation coefficient, whereas panel (b) shows the retrieved amplitude factor, both as a function of the vial’s depth. The behavior of κ indicates that the combination of source–detector separation used in this study maximizes the sensitivity of the system at a depth of inclusion equal to 18.5 mm. To see this, we could imagine a scenario where there is no ICG in the vial, in which case κ should be independent of its depth, with the consequence that the fitted values should present a completely flat and horizontal behavior around $\kappa ∼0.12\;{\mathrm{mm}}^{-1}$; the fact that this value changes with the depth of the vial makes us believe that the system is detecting its presence as it goes deeper and deeper into the medium (as least up to a depth of 18.5 mm). This trend can be explained as follows: although the excitation photons (i.e., photons coming from the source at $\lambda_{x}$) do not encounter the object in their trajectory from source to detector, the retrieved effective attenuation coefficient resembles the one from the surrounding liquid phantom, i.e., $\kappa ={\left(3\mu_{aM}\mu_{s^{\prime }}\right)}^{-1/2}$; on the other hand, when the excitation photons begin to sense the vial (at a depth between 8.5 and 10.5 mm), more emission photons are produced, and hence, κ increases because the absorption coefficient tends to be the sum of $\mu_{aM}$ and $\mu_{af}$.^24^^,^^25^ Beyond 18.5 mm, the value of κ significantly decreases, although not to the baseline level, suggesting that deeper inclusions could still be detectable by the measurement system. Regarding the baseline level, the chosen optical properties for the host medium ($\mu_{aM}=0.01\;{\mathrm{mm}}^{-1}$ and $\mu_{s^{\prime }}=1\;{\mathrm{mm}}^{-1}$) should give a value of $\kappa ∼0.17\;{\mathrm{mm}}^{-1}$. As it can be seen from the first retrieved value in Fig. 5(a), this is not the case; this difference could be attributed to errors during the phantom preparation or to an inaccurate choice of the model from Ref. 21.

The exponential decay of the amplitude factor C in Fig. 5(b) is probably due to the fact that this parameter is a function of the product between $\mu_{af}$ and η [as expressed by Eq. (3)]. Hence, as the object moves further away from the source and detector, the liquid phantom without ICG dampens the absorption and the quantum yield of the ICG solution in the vial, with the consequent impact on C just discussed. Here, we must stress that we are using a homogeneous model to test a heterogeneous medium, so what the model actually retrieves is an “effective” absorption coefficient of the fluorophore together with an “effective” quantum yield, which will vary according to the position of the object. This claim is further supported by the form of C given in expression Eq. (3) derived above.

According to these results, the sensitivity of the system could be possibly extended to depths larger than 20 mm; nevertheless, a more thorough study must be performed, for example, by varying the optical properties of the host phantom and the size of the inclusion, or by refining the model used to fit the experimental data.

## 4 *In vivo* Animal Studies

The fluorescence depth sensing system was compared with the EleVision IR platform (Medtronic, Minneapolis, Minnesota, United States), a clinically available surface fluorescence imaging system used for NIR monochromatic imaging of superficial tissues. The imaging distance between the camera and the subject was set at 30 cm for all imaging sessions, which was within the 25- to 55-cm focal depth of the camera. A radiometric target (QUEL Imaging LLC, White River Jct, Vermont, United States), an adjustable solid-state emitter that mimics the emission wavelength of ICG, was placed in the field of view for all trials. The output of this target is adjusted by changing the voltage of the power source, so injecting a test mouse with ICG is a necessary step in determining the proper voltage output before imaging the experimental cohort.

Each mouse received two identical doses of ICG. Immediately after the first injection, which was used for imaging with the fluorescence depth sensing system, the mouse would receive another ICG injection for imaging with the EleVision system. NIR monochromatic images were acquired at 2-s time intervals for the first minute of imaging to capture the fast ICG uptake kinetics, and at 15-s time intervals for the remaining 9 min to capture the slower kinetics of ICG clearance. Regions of interest for the tumor, normal tissue, and radiometric target were selected by hand using ImageJ, and the mean intensity of each of them was acquired for each image.

**Fig. 6:**
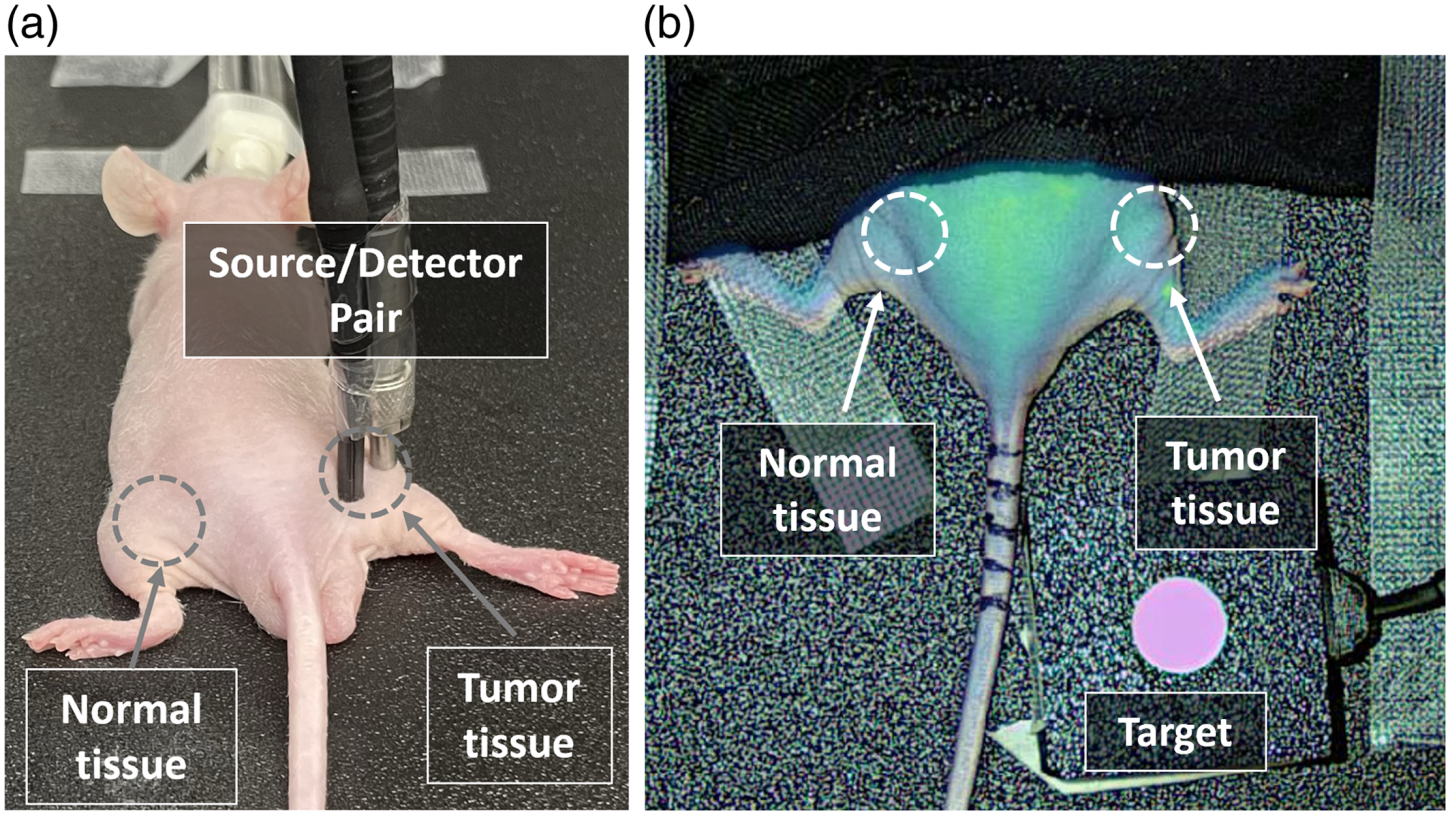
*In vivo* imaging of a mouse with (a) subsurface fluorescence measurement at a 5-mm source-detector separation and (b) surface imaging by the EleVision system. The radiometric target shown in panel (b) was kept in the field of view for all images and used to compensate f.

Using this fluorescence depth sensing system, *in vivo* imaging of four mice was carried out using the source–detector pair to record the fluorescence intensity over time for 10 min on both normal and tumor tissues for each mouse. Here, the imaging time was chosen to include the rapid transient uptake of ICG kinetics in the initial few seconds after injection, with a peak signal of ICG followed by a gradual decrease from plasma clearance kinetics. Figure 6(a) illustrates the geometry of *in vivo* imaging using a source–detector pair measured on tumor and normal tissues using fluorescence depth sensing. The laser modulating frequency was at 2 Hz, and the measured output laser power from the fiber optic was ∼62  mW. Furthermore, the magnitude of the voltage signal was maintained well below the threshold saturation at 10 V, and the laser intensity used was at the maximum range by applying a laser set current at 110 mA. Furthermore, as a comparative study, the surface EleVision system was used for the *in vivo* imaging of mice on tumor and normal tissues for uptake and clearance kinetics. Figure 6(b) illustrates the prototype *in vivo* imaging of mice using a surface imaging system.

### 4.1 Tracking ICG Kinetics on Different Days with Increasing Tumor Size

**Fig. 7:**
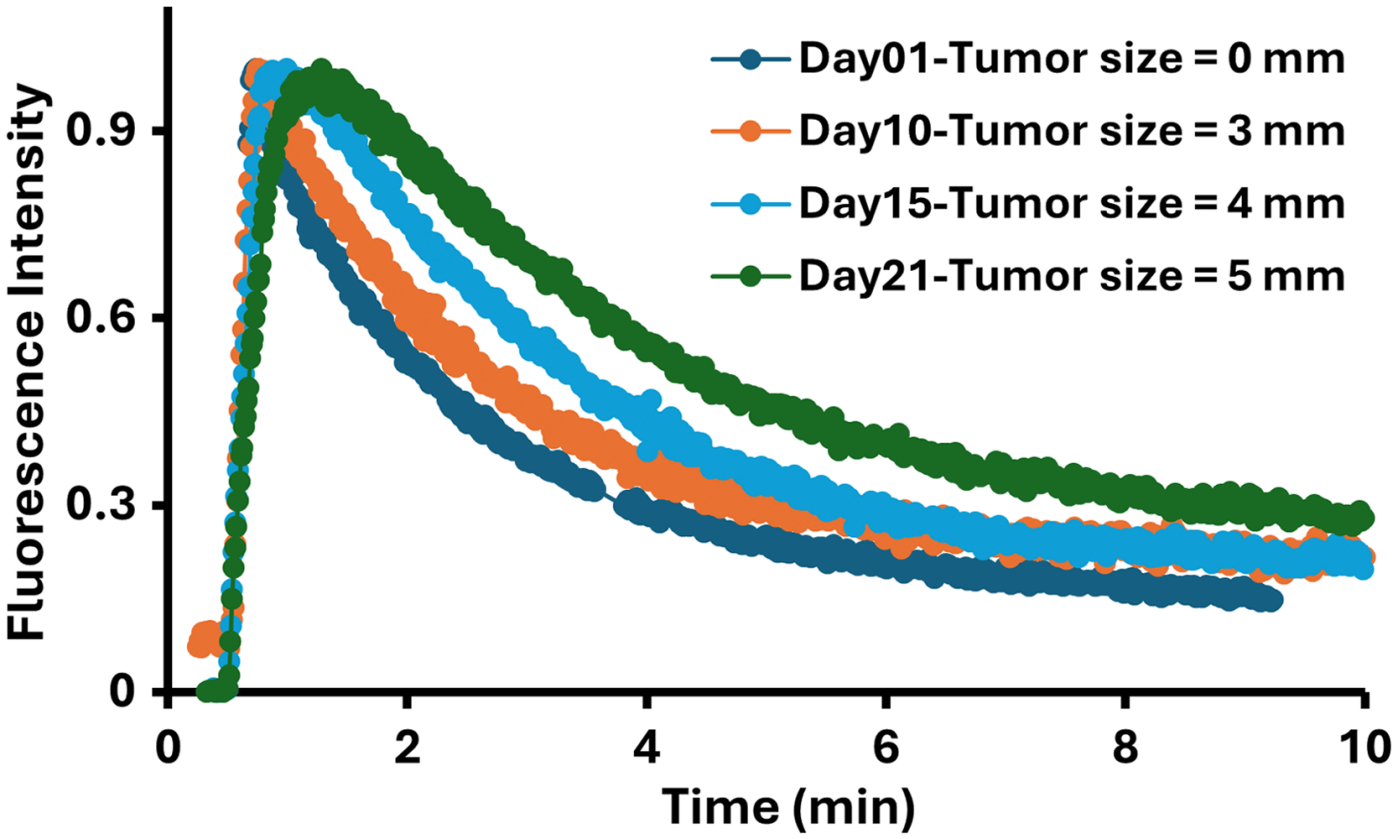
Fluorescence depth sensing system was used to track ICG kinetics for 10 min in each mouse at multiple time points, which corresponded to different tumor sizes. Analysis showed that the system is able to resolve kinetic differences as a tumor becomes larger, with larger tumors showing delayed uptake and longer retention of ICG or the system autogain.

During this study, fluorescence depth sensing was used to image through the skin and into the tumor. The ICG kinetic curve for normal tissue had a very rapid uptake and fast clearance with a bi-exponential clearance curve, whereas the tumor was expected to be slower in both. This was imaged for successive days after tumor implantation, including days 0, 10, 15, and 21 with gradual growth of tumor size measured at 0, 3, 4, and 5 mm, respectively, on these days. For all of the *in vivo* imaging experiments here, the source–detector pair was positioned 5 mm apart located gently on the surface of both the legs of the mouse, and the background light was suppressed by a black covering. Furthermore, the obtained data points were normalized to have the tail regions match both tumor and normal tissues. Figure 7 illustrates the ICG kinetics sampled on four mice on different days with increasing growth of tumor size wherein all the peak values were normalized to 1 for better comparison, analysis, and processing. From this, it is clear that the tumor tissue uptake and clearance are slow in the kinetics curve and vary based on the tumor tissue sampled.^25^

### 4.2 Fluorescence Depth Sensing Versus Surface Imaging System for ICG

**Fig. 8:**
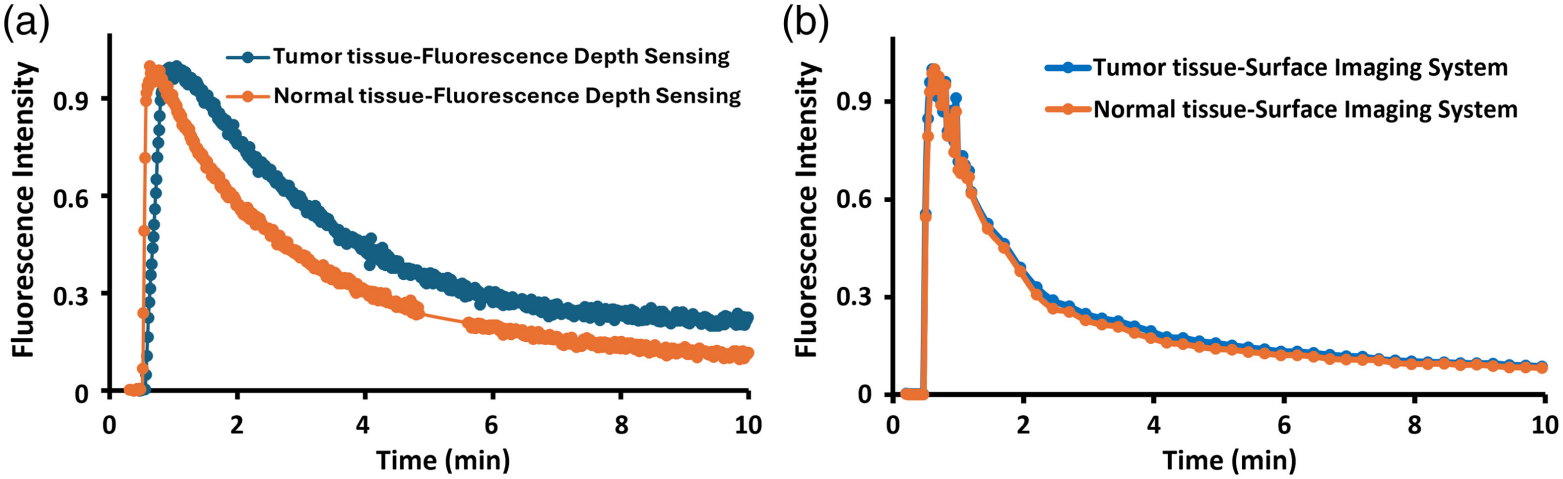
Comparison of ICG kinetics in normal and tumor tissues sampled on the (a) fluorescence depth sensing system and (b) surface imaging system. The deeper imaging of the fluorescence depth sensing system allows it to better resolve kinetics differences between the tissue types.

The radiometric target served as a reference to compensate for the EleVision system’s autogain function so that we could normalize the ICG curves. Normalizing to an ICG-equivalent reference target has been previously validated.^26^ These normalized ICG curves of both the tumor tissue and the normal tissue were compared with the curves obtained by the depth sensing approach to compare the ability of the systems to observe kinetic differences in the ICG kinetics between the tissue types. Usually, surface imaging is highly weighted to the most superficial tissues, i.e., skin layer, and cannot penetrate deep within the tissue and provide much information beyond the superficial tissues. Thus, this is one of the major drawbacks of the surface imaging system in the field of optical imaging and restricts it from being used for high penetration depth studies, especially beneath superficial tissue. Figure 8 shows the ICG kinetics for tumor and normal tissues imaged on the surface of the mice using (a) a depth sensing system and (b) a surface imaging system.

### 4.3 Analysis of ICG Kinetics

Based on the measured slopes of the ICG signal with fluorescence-to-excitation intensity ratio, kinetic modeling simulations were conducted to promote the hypothesis that feature tumor pathophysiology, such as capillary or vascular leakage, could be responsible for the observed differences across tumor types. Fitted uptake and clearance kinetics show that tumors are slower, both in uptake and clearance. Furthermore, the obtained data points from all the investigated days, both tumor and normal tissues, were taken into consideration, and the data were interpreted to fit the modeling theory of ICG kinetics. The ICG kinetics included two exponential curves, including the rising and falling parts over time for pre-peak and post-peak, respectively, fitting to exponential-type equations for separate parts of the rising and falling parts of the kinetics. It was noticed that the falling off curve, after the peak, was a bi-exponential functional form, fitted using Eq. (4)

y(t)=A*exp(−B*t)+C*exp(−D*t) (4)

where A, B, C, and D are free-fit curve parameters with exponential decay function with respect to time, t. Similarly, the trailing part of the rising curve, before the peak, was fitted to a function that is exponential saturating in nature using Eq. (5).

$$z(t)=(1-\exp(-M*(t-t_{p}))$$ (5)

where M is a free-fit parameter and $t_{p}$ is the time-to-peak. Table 1 summarizes the condensed curve fit parameters for both tumor and normal tissues by taking the average values of these free-fit parameters.^27^ Here, for the tumor tissue, it can be seen that each of them has slower uptake and clearance, and for the normal tissue, the rise and fall time will be faster.

**Table 1:** Curve fit parameters for rising and falling parts of the ICG kinetics.

Tissue and day	Falling curve (post-peak)				Rising curve (pre-peak)	
	A	B ($\min^{-1}$)	C	D ($\min^{-1}$)	M ($\min^{-1}$)	$t_{p}$ (min)
Tumor tissue day 10	0.80	0.56	0.18	0.024	11.8	0.052
Tumor tissue day 15	0.87	0.35	0.17	0.036	12.1	0.048
Tumor tissue day 21	0.98	0.25	0.12	0.043	12.3	0.075
Normal tissue average	1.01	0.33	0.15	0.042	11.2	0.059

At this point, it must be noted that we lack a physiological model to compare with the experimental data, and hence, no physiological parameters are retrieved in this fitting process. Instead, a semi-quantitative analysis was performed to notice the fast uptake and clearance of the kinetics curves, which easily differentiates between tissue types. This is a common practice, and although quantitative physiological parameters cannot be studied, a qualitative behavior can be inferred by analyzing the exponential constants.^28^

### 4.4 Fluorescence Depth Sensing Versus Wide Field Imager System for PpIX

Herein, a comparison was made for PpIX imaging using a laboratory setup, which utilizes a 650-nm long pass-filtered color camera for capturing PpIX emission. The wide-field imager collects at an 8×8  cm field of view (FOV) covering the body of the mouse. Excitation of PpIX is accomplished through a 635-nm LED with a power density of $5\;\mathrm{mW}/{\mathrm{cm}}^{2}$ in the FOV. PpIX prompt fluorescence is measured at an overall integration time of 50 s. Acquisition parameters are kept the same across the measurements.

After ALA injection, four mice were imaged with both a fluorescence depth sensing system and a wide-field imager. Mice were imaged at time points of 1-, 3-, and 6-h post-5-ALA injection, with limited sampling due to the need for re-anesthesia each time. For control, images and measurements were also acquired before 5-ALA administration and right after 5-ALA administration.

**Fig. 9:**
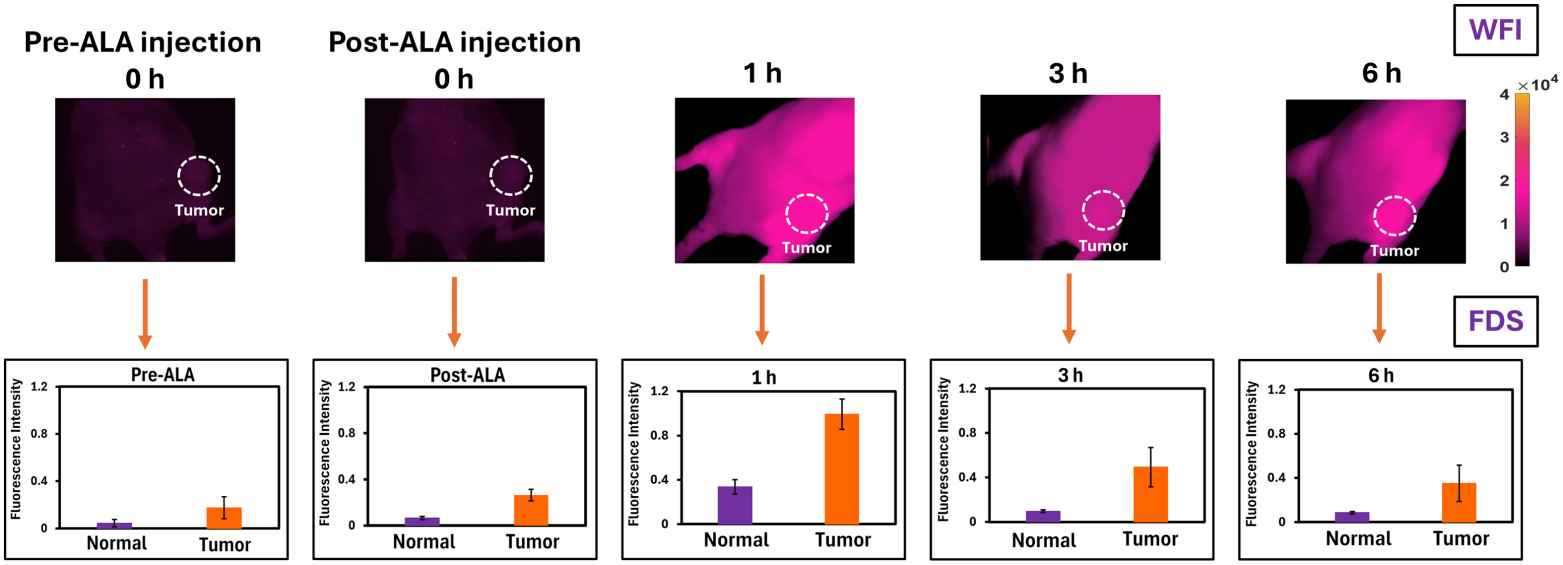
Both wide-field imager (WFI) and fluorescence depth sensing (FDS) systems were used to measure PpIX fluorescence in both tumor and normal tissues at multiple time points after 5-ALA administration. The measurements taken by the fiber optic-based FDS system correlated with the images of the wide-field imager, which showed stronger fluorescence in tumor signal in the later time points and fluorescence peaking at 1-h post-injection.

For measurements, mice were under isofluorane-based anesthesia, and measurements were acquired first with the wide field imager and subsequently with the fluorescence depth sensing system for both tumor and non-tumor thighs. For quantification of the wide-field images, ROIs are selected at both the tumor and opposite leg regions of the mouse, and mean and standard deviation are calculated per each time point. Example fluorescence images acquired at the different time points for one mouse are displayed in Fig. 9 in correlation to the fluorescence depth sensing system measurement quantification.

**Fig. 10:**
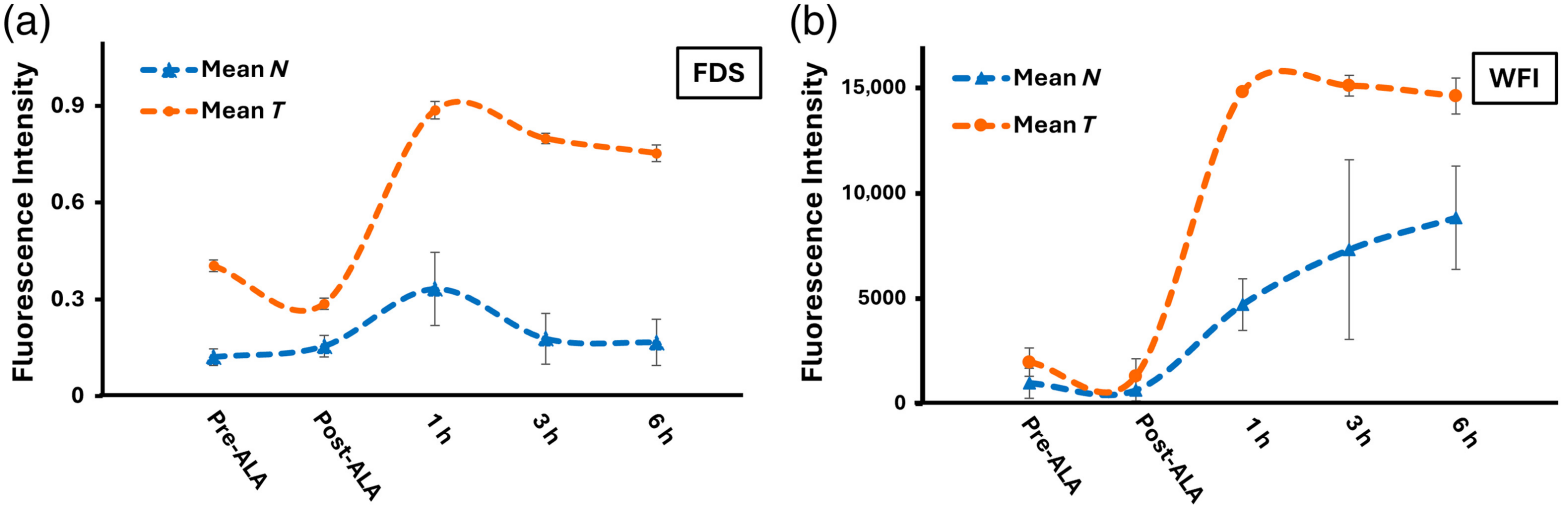
Mean PpIX fluorescence for both tumor (mean T) and normal (mean N) tissues measured with (a) the fluorescence depth sensing (FDS) and (b) the wide field imager (WFI) system both before 5-ALA injection and up to 6 h after injection.

The results shown in Fig. 10(a) describe an analysis obtained through fluorescence depth sensing, and Fig. 10(b) displays images acquired with the PpIX wide-field imager. When calculating the mean and standard deviation of the group, as measured with the fluorescence depth sensing system and wide-field imager [Figs. 9(a) and 9(b)], it can be observed that the overall trend correlates with the acquired fluorescence intensity. However, an exact matching of results is not expected as the fluorescence depth sensing system samples a smaller region of interest in comparison to the wide-field imager, where the ROIs cover the whole tumor and a similar area on the opposite side of the mouse. In comparison to ICG, the kinetics of PpIX are much slower with both systems as described as a significant change was not observed within a single time point. Notably, the detected PpIX with both systems can be a combination of both PpIX skin fluorescence and PpIX accumulation in the tumor.

## 5 Discussion

The significance of the fluorescence depth sensing system for *in vivo* imaging of ICG and PpIX is to measure and monitor capillary permeability and porphyrin production. Herein, to study surface fluorescence kinetics, including clearance and uptake of normal and tumor tissues through ICG and PpIX fluorophore, this system was developed. The capability of this system was investigated and validated here both experimentally and theoretically. Also, a comparison study was carried out between the fluorescence depth sensing system and surface imaging systems to show the differences in observed kinetics and to distinguish the temporal signals that originate in the tumor versus normal tissues.

In most commercial imaging systems, the imaging is limited to superficial tissues, with the depth of sensitivity being difficult to specify exactly and varying with different systems.^29^ Imaging more superficial regions of mice rather than with deeper ones limits the ability of planar imaging systems to truly provide a signal that has complete value to represent the tumor. Planar imaging systems work well for invasive surgical procedures where the purpose is to remove tissue and expose the tissue to be examined/tested, but their use in pre-clinical subcutaneous tumors has always had this limitation of the signal from the overlying normal skin. Thus, there is value in the development of a fluorescence depth sensing system that can measure through the skin to access deeper sensing down to perhaps 20-mm depth, as shown in Fig. 4(b). The data of ICG kinetics in Fig. 8(a) highlight the value of sampling through the tissue versus off the surface because the slow vascular kinetics of the tumor are visible in the curve. The classic shape of contrast kinetics through tumors is a slower uptake time and a slower clearance time, and this shape is seen as the tumor grows (Fig. 7). Surface imaging would not provide any of this tumor sensitivity kinetic data, as shown in Fig. 8(b).

A secondary goal of the current investigation was to develop and test a cost-effective dual-channel prototype system that can quantify both ICG and PpIX concentrations through the skin by designing the filters and light sources so that no moving parts are required to switch between these signals. The various phantom and mouse tests accomplished in this study revealed that the system worked well, and showed the potential in several pilot studies.^30^^,^^31^ The proposed design had a two-detector channel approach where the excitation and fluorescence detectors simply passively switch when the light source is switched. So, by electronically turning on the source for ICG or PpIX, the two detectors implicitly sense the optical signals, without any hardware movements for sequential measurements of these fluorophores.^32^^–^^34^ Based on the results, we have proven that the design can work to measure fast ICG kinetics and slower PpIX kinetics with the same detection hardware, adding novelty to this proposed study.

In the future, this system will be extended to a tomographic approach with several sources and detectors and also incorporated with an ultrasound transducer probe in between the sources and detectors creating a gap of ∼20  mm, so the localization of the signal could be achieved with ultrasound, and the functional aspects of the tumor tissue could be sensed with the fluorescence channel. The data in this study show the potential of the system that even with a maximum distance between the source and detector apart, it was able to capture the ICG data points with slight variation in the background noise that was removed by applying the data analysis filters to smoothen the kinetics curve. In addition, with this source–detector separation, we should be able to potentially sense 20-mm deep (see Fig. 4) to see the temporal kinetics of these two contrast agents.

This paper has limited *in vivo* data, but the major goals of the study were technical to demonstrate and confirm the value of fluorescence sampling via deeper sensing and second to show that two fluorophores could be sampled passively with the same detector circuit. Further biological studies will follow as the system is refined, but this technical feasibility study presents the core functioning and design necessary to move forward with more biological work.

## 6 Conclusion

In this work, the design and performance of a dual-band fluorescence depth sensing system have been highlighted and validated by systematic phantom studies and *in vivo* imaging of mice, using tumor growth and comparisons between normal and tumor tissues to show temporal kinetics differences in ICG and PpIX signals. Deep tissue sensing of tumor ICG kinetics shows expected uptake and clearance curves that match expected values, with both uptake and clearance times progressively being slower with tumor growth. These deep ICG and PpIX kinetic curves were compared with the standard benchtop surface imaging system and wide-field imaging system, which shows temporal kinetics that was nearly completely dominated by the overlying surface tumor tissue. Thus, this depth-sensing approach has a lot of value in tracking tumors over time and in response to therapy studies. Basic phantom investigations were conducted to characterize features of sensitivity, stability, and depth penetration, suggesting that sensing as deep as 20 mm into tissue may be possible. Furthermore, the unique detector design of this system makes it possible to passively sense signals from either ICG or PpIX, without any hardware movements in the detection channel and by just exciting at either 780 or 633 nm, respectively. The fluorescence-to-excitation ratio minimizes problems of fiber coupling and tissue excitation fluctuations. This prototype system is expected to provide a cost-effective approach to the future direction in the development of a multichannel optical imaging array coupled with ultrasound imaging for tracking tumor responses to interventional therapies.

## Data Availability

Data and code developed in this paper are available upon reasonable request to the corresponding author.
